# The potential of pregnant women as a sentinel population for malaria surveillance

**DOI:** 10.1186/s12936-019-2999-0

**Published:** 2019-11-21

**Authors:** Nina C. Brunner, Frank Chacky, Renata Mandike, Ally Mohamed, Manuela Runge, Sumaiyya G. Thawer, Amanda Ross, Penelope Vounatsou, Christian Lengeler, Fabrizio Molteni, Manuel W. Hetzel

**Affiliations:** 1 Swiss Tropical and Public Health Institute, Socinstrasse 57, 4002 Basel, Switzerland; 2 University of Basel, Petersplatz 1, 4003 Basel, Switzerland; 3 National Malaria Control Programme, P.O. Box 9083, Dar es Salaam, United Republic of Tanzania; 4 Ministry of Health, Community Development, Gender, Elderly and Children, Building No. 11, P. O. Box 743, 40478 Dodoma, United Republic of Tanzania

**Keywords:** Malaria, Surveillance, Malaria elimination, Pregnant women

## Abstract

**Background:**

With increasing spatial heterogeneity of malaria transmission and a shift of the disease burden towards older children and adults, pregnant women attending antenatal care (ANC) have been proposed as a pragmatic sentinel population for malaria surveillance. However, the representativeness of routine ANC malaria test-positivity and its relationship with prevalence in other population subgroups are yet to be investigated.

**Methods:**

Monthly ANC malaria test-positivity data from all Tanzanian health facilities for January 2014 to May 2016 was compared to prevalence data from the School Malaria Parasitaemia Survey 2015, the Malaria Indicator Survey (MIS) 2015/16, the Malaria Atlas Project 2015, and a Bayesian model fitted to MIS data. Linear regression was used to describe the difference between malaria test-positivity in pregnant women and respective comparison groups as a function of ANC test-positivity and potential covariates.

**Results:**

The relationship between ANC test-positivity and survey prevalence in children follows spatially and biologically meaningful patterns. However, the uncertainty of the relationship was substantial, particularly in areas with high or perennial transmission. In comparison, modelled data estimated higher prevalence in children at low transmission intensities and lower prevalence at higher transmission intensities.

**Conclusions:**

Pregnant women attending ANC are a pragmatic sentinel population to assess heterogeneity and trends in malaria prevalence in Tanzania. Yet, since ANC malaria test-positivity cannot be used to directly predict the prevalence in other population subgroups, complementary community-level measurements remain highly relevant.

## Background

Malaria surveillance has been receiving increasing attention, with the World Health Organization (WHO) recognizing surveillance as a core intervention in its Global Technical Strategy for Malaria 2016–2030 [1]. Currently, different approaches are used to assess morbidity, mortality, and transmission of malaria and monitor trends over time in endemic countries. Two major sources of malaria data are health management information systems (HMIS) and nationally representative household surveys such as the Demographic and Health Surveys (DHS) and Malaria Indicator Surveys (MIS). However, while case-based surveillance data reported through the HMIS only reflect clinical cases accessing formal health facilities, household surveys are too expensive to provide real-time data for continuous malaria surveillance. Several countries are therefore implementing additional data collection strategies, such as sentinel surveillance sites or school surveys [2–5]. To complement these efforts, pregnant women attending antenatal care (ANC) services have been proposed as a pragmatic sentinel population for the surveillance of community-level malaria prevalence [6]. Pregnant women data can be routinely collected relying on existing infrastructure and mechanisms, thus, providing a cost-effective and real-time estimate of malaria prevalence.

The relationship between the malaria prevalence in pregnant women and children aged 0–59 months has previously been investigated in a systematic review and meta-analysis which found a strong correlation between both groups [7]. Yet, only one study recruiting participants from an antenatal clinic was included in the meta-analysis. Additionally, the authors made a pooled analysis of prevalence data obtained from different administrative levels which made it impossible to give any recommendations on what spatial scale ANC prevalence might be used to monitor malaria transmission. Another study by Hellewell et al. found that clinical malaria incidence in children can predict ANC prevalence up to 3 months in the future but not vice versa [8]. However, data from only five hospitals were analysed and no uniform relationship between prevalence and incidence could be established. Therefore, the validity of large-scale routine ANC prevalence data for monitoring transmission in children has yet to be investigated.

This study contributes to the existing evidence by examining the agreement between nationwide routine ANC test-positivity data and malaria prevalence in children at different administrative levels.

## Methods

### Data source and data cleaning

The mainland Tanzania’s District Health Information System 2 (DHIS2) was accessed on 4th July 2016 and the monthly number of first ANC attendances, re-attendances, the number of malaria tests performed, as well as the number of positive malaria tests by health facility were downloaded for the period January 2014 to May 2016. Additionally, a variable indicating successful submission of the monthly electronic report was obtained to calculate reporting rates.

As the DHIS2 database is unable to store numbers with the value zero, missing values of numerical variables were replaced with zero in case the reporting variable indicated successful form submission. Data cleaning of non-zero values was based on assumptions taking into account the relation between the indicators and the distribution of the data. Specifically, reports with malaria positivity above 100% were considered invalid. However, malaria testing rates above 100% were acceptable to a certain limit depending on the number of re-attendances as some women may be tested during a follow-up visit. Values identified to be inconsistent, invalid or outliers were set to missing. Because only single values instead of whole reports were deleted, the number of reports and, consequently, the number of malaria tests included in the calculations of the proportion of women tested and the calculation of the malaria test-positivity were different (Additional file 1: Figure S1).

### Descriptive analysis

The number of malaria tests as a proportion of the number of first ANC attendances was calculated as a proxy for the proportion of women receiving a malaria test. The malaria test-positivity was obtained by dividing the number of positive malaria tests by the number of tests performed. The 95% confidence intervals for malaria test-positivity at district and regional level were calculated adjusting for clustering at health facility level using a clustered sandwich estimator. Facility reporting rates defined as the percentage of monthly electronic reports successfully submitted to the DHIS2 were evaluated for the year 2016 only as the list of health facility is continuously updated in the DHIS2, thus, changing the denominator with every new facility formally registered in the system. The reporting rates for 2016 were calculated based on the assumption that the number of health facilities did not increase substantially during the first 5 months of the year. All statistical analyses were conducted in STATA version 14 (StataCorp LP, Texas, USA).

### Comparative analysis

ANC test-positivity data was aggregated on common administrative levels (region, district, health facility) to be compared with available prevalence data (Table 1). Changes in the administrative division of Tanzania over time required adapting the number of districts in the ANC dataset to the number in the comparison dataset. To adjust for seasonal variation, the selection of ANC data was restricted to the same observation period as the comparison data resulting in a different ANC sample size for each comparison.

**Table 1:** Characteristics of studies included in the comparison of malaria prevalence in children versus ANC test-positivity, Tanzania, 2014–2016

Data source	Observation period	Design	Level of information	Sample size	Age	Comparative ANC sample size
ANC	January 2014–May 2016	HMIS	Health facility (6187) District (178) Region (25)	2,190,877	Reproductive age	NA
SMPS 2015 [9]	August 2014, September 2014, May 2015, October 2015	Survey	District (166)	48,445	5–16 years	82,020
TDHS-MIS 2015/16 [10]	August 2015–February 2016	Survey	Region (25)	8847	6–59 months	297,967
MAP 2015 [12]	2015	Model	District (176)	NA	2–10 years	1,033,217
BGM	2015	Model	Health facility (5612)	NA	6–59 months	1,033,217

*SMPS* School Malaria Parasitaemia Survey, *TDHS-MIS* Tanzania Demographic and Health Survey and Malaria Indicator Survey, *MAP* Malaria Atlas Project, *BGM* Bayesian geo-statistical model, *NA* not applicable

All published raw and model-based prevalence data at national level for the time period January 2014 to May 2016 was considered. Available primary data included the school malaria parasitaemia survey 2015 (SMPS) and the Demographic and Health Survey and Malaria Indicator Survey 2015/16 (TDHS-MIS) [9, 10]. The Malaria Atlas Project (MAP) district prevalence estimates for 2015 were extracted with RStudio v1.0.136 (R Foundation for Statistical Computing, Austria) using a shapefile provided by the National Malaria Control Programme of Tanzania and applying population weighting using population densities obtained from the Worldpop website [11, 12]. In addition, the analysis included a comparison with more direct estimates of a Bayesian geo-statistical regression model (BGM) fitted to the MIS 2015/16 RDT results without adjustments for age, time, and test sensitivity. The BGM was computed using the methodology described by Ssempiira et al. [13]. To approximate the health facility catchment area, Voronoi polygons were drawn around health facilities with available geo-coordinates and prevalence predictors, and malaria prevalence was extracted. No population weighting was applied under the assumption that the Voronoi polygon areas are acceptably homogeneous and, therefore, less prone to bias.

The relationship between the malaria test-positivity in pregnant women and the prevalence in the respective comparison group was assessed using an approach based on the methods for assessing agreements suggested by Bland and Altman [14]. This method estimates the overall bias between groups, and the variability in differences in prevalence for individual areas. First, the difference between the malaria test-positivity in pregnant women and the prevalence in the respective comparison group was plotted against the ANC test-positivity. Because the difference varied with increasing ANC test-positivity and the relationship could not be removed by log transformation, the test-positivity difference was regressed on the ANC test-positivity. Covariates were added to the regression model to investigate whether the relationship was altered in the presence of different factors. Independent variables that were considered included seasonality after stratification by geographic zone used in the TDHS-MIS 2015/16 (Additional file 1: Table S1), insecticide-treated net (ITN) coverage in the comparison group, and level of urbanization by stratifying according to the type of district council (district comparisons only). ITN coverage was centred at the respective mean value. Municipal and city councils were classified as urban, township councils as semi-urban and district councils as rural. A covariate was included in the multivariable model if the effect size estimate was significant at a level of 0.2 in the baseline model including the prevalence difference as outcome and ANC test-positivity as predictive variable. A covariate remained in the multivariable model if it was significantly associated with the outcome on a 5% confidence level. Additionally, it was tested whether the covariates were effect modifiers of the relationship by testing interaction terms. Again, the interaction term was included in the multivariable model if it was significant at a level of 5%. All models were adjusted for clustering on aggregation level using a clustered sandwich estimator.

Some of the plots showed that the variability of the difference changed in relation to the ANC test-positivity. Following the suggestion of Bland and Altman, the absolute residuals of the previously fitted regression lines were thus modelled as a function of the ANC test-positivity to obtain the 95% limits of agreement [14]. The regression models for the 95% limits of agreement included the same covariates as the regression model for the mean difference.

## Results

### Analysis sample

In total, 6187 health facilities out of 7638 (81.0%) reported monthly ANC indicators between January 2014 and May 2016 at least once. Assuming that all 6187 health facilities were operational for the entire time period, the theoretical number of monthly reports is 179,423. After excluding invalid or missing reports and values, the malaria testing rate was calculated based on 150,424 reports (83.8%) and the malaria test-positivity was obtained based on 102,727 reports (57.3%). A detailed description of the number of reports included in the statistical analysis is shown in Additional file 1: Figure S1.

### ANC reporting rate

Between January 2014 and May 2016, 164,625 of 179,423 reports (91.8%) were submitted to the DHIS2. In 2016, the average monthly reporting rate was 96.5% (N = 30,935). A marginally lower reporting rate in urban districts was related to a high proportion of private health facilities (57.5%) which tended to have a lower reporting rate (85.8%, N = 1645) than public (97.5%, N = 25,480) and faith-based institutions (96.3%, N = 3240) independent of district type.

### ANC malaria testing

Overall, 4,354,911 first ANC consultations and 2,161,301 (49.6%) malaria tests were reported. The number of malaria tests in relation to the number of first attendances more than doubled from 30.1% in January 2014 to 71.3% in May 2016. The proportion of women tested was higher in urban areas (61.7%) compared to semi-urban (49.6%) and rural areas (46.6%) with a difference that was stable over time. Additionally, the testing proportion in districts with average malaria prevalence in pregnant women of less than 5% between January 2014 and May 2016 was 59.9% compared to 42.5% in districts with more than 5% malaria prevalence. The difference remained after stratifying for district type and was consistent over time (Additional file 1: Figure S2).

### Prevalence comparison

#### School malaria parasitaemia survey 2015

**Fig. 1:**
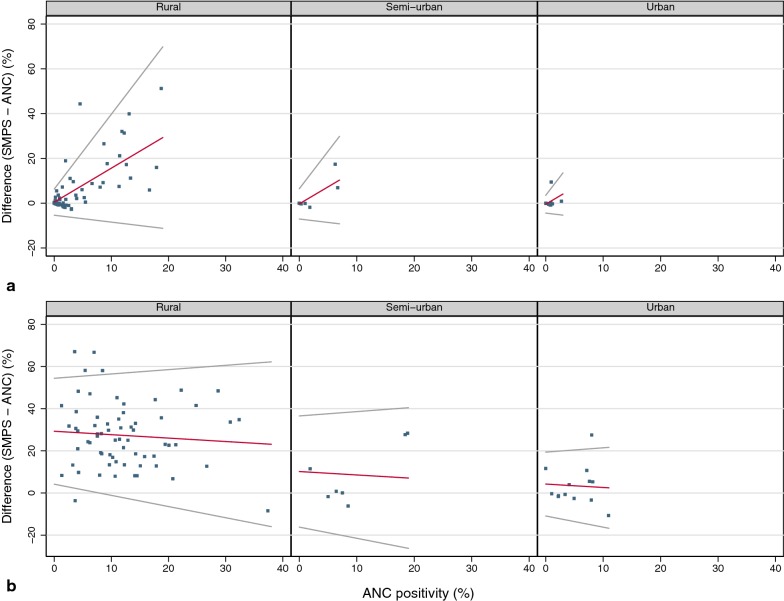
District test-positivity difference SMPS − ANC against ANC malaria test-positivity with regression line and 95% limits of agreement, normalized for ITN use in school children. **a** Districts with seasonal malaria transmission, by district type. **b** Districts with perennial malaria transmission, by district type

The national malaria test-positivity in pregnant women during the time of the SMPS was on average lower than in school children [6.4% (95% CI 5.9–6.9) vs. 21.7% (95% CI 19.6–23.9)]. In the district comparison, seasonality, urbanization, and ITN usage in school children were significant covariates. In districts with seasonal malaria transmission, the regression model predicted a monotonically increasing test-positivity difference with ANC test-positivity irrespective of level of urbanization (Fig. 1a, Table 2). In districts with perennial malaria transmission, the difference did not change substantially with varying ANC test-positivity but decreased from rural to semi-urban and urban districts (Fig. 1b, Table 2). The use of ITNs only marginally increased the test-positivity difference by 1.5% (95% CI 0.3–2.7) for an increase of ITN usage of 10%.

**Table 2:** Modelled test-positivity difference with 95% limits of agreement between children and pregnant women attending ANC at different levels of ANC test-positivity

ANC test- positivity (%)	SMPS^a^						MIS	MAP	
	Seasonal			Perennial				Seasonal	Perennial
	Rural	Semi-urban	Urban	Rural	Semi-urban	Urban			
0	0.48 (− 5.37; 6.33)	− 0.29 (− 7.05; 6.47)	− 0.45 (− 4.41; 3.51)	29.30 (4.17; 54.43)	10.18 (− 16.19; 36.56)	4.24 (− 10.91; 19.39)	− 0.29 (− 1.39; 0.81)	4.83 (1.94; 7.72)	6.36 (− 0.65; 13.38)
5	8.07 (− 6.91; 23.04)	7.29 (− 8.59; 23.18)	7.13 (− 5.95; 20.22)	28.49 (1.52; 55.45)	9.37 (− 18.84; 37.58)	3.42 (− 13.56; 20.41)	3.20 (− 4.98; 11.39)	0.56 (− 2.43; 3.56)	2.10 (− 5.02; 9.21)
10	15.66 (− 8.45; 39.76)	14.88 (− 10.13; 39.89)		27.67 (− 1.13; 56.48)	8.56 (− 21.49; 38.61)	2.61 (− 16.21; 21.44)	6.70 (− 8.57; 21.96)	− 3.70 (− 6.8; − 0.61)	− 2.17 (− 9.39; 5.05)
15	23.24 (− 9.98; 56.47)			26.86 (− 3.78; 57.50)	7.74 (− 24.14; 39.63)		10.19 (− 12.15; 32.54)	− 7.97 (− 11.17; − 4.77)	− 6.44 (− 13.76; 0.88)
20	30.83 (− 11.52; 73.18)			26.05 (− 6.43; 58.53)	6.93 (− 26.79; 40.65)		13.69 (− 15.74; 43.11)	− 12.24 (− 15.54; − 8.94)	− 10.71 (− 18.13; − 3.28)
25				25.23 (− 9.08; 59.55)				− 16.51 (− 19.91; − 13.1)	− 14.98 (− 22.5; − 7.45)
30				24.42 (− 11.73; 60.57)					− 19.24 (− 26.87; − 11.61)
35				23.61 (− 14.38; 61.60)					
40				22.80 (− 17.03; 62.62)					

The 95% limits of agreement represent the range in which 95% of the individual school children prevalence − ANC test-positivity differences are expected to lie

^a^Normalized for ITN usage in school children

There was a wide uncertainty around the relationship. The variability was lowest for districts with seasonal transmission at low ANC test-positivity. With increasing ANC test-positivity, however, the relationship became less predictable. For districts with perennial transmission, the 95% limits of agreement were considerably wide at any level of ANC test-positivity.

#### Demographic and Health Survey and Malaria Indicator Survey 2015/16

**Fig. 2:**
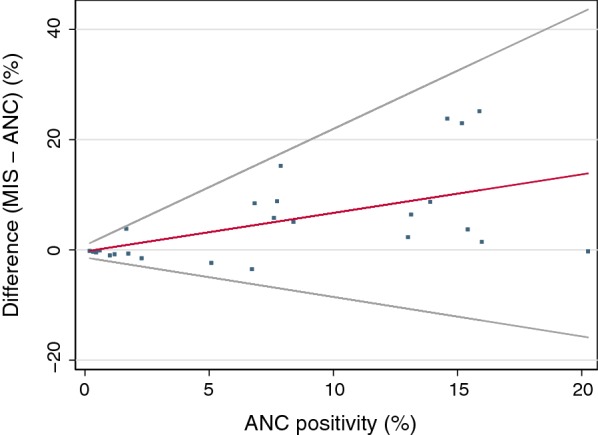
Regional test-positivity difference MIS − ANC against ANC malaria test-positivity with regression line and 95% limits of agreement

The TDHS-MIS 2015/16 found a national malaria prevalence of 14.4% (95% CI 12.5–16.4) for Mainland Tanzania in children aged 6–59 months. The corresponding test-positivity in pregnant women attending ANC was 7.2% (95% CI 6.8–7.6). At regional level, the test-positivity difference increased with increasing ANC test-positivity and was not influenced by seasonality or ITN use. The difference at low ANC test-positivity levels was negligible and there was a tendency of the prevalence in young children being lower than in pregnant women up to an ANC test-positivity of around 5% (Fig. 2, Table 2). The variability at low ANC test-positivity was small but increased substantially for higher transmission levels.

#### Malaria Atlas Project estimates for 2015

**Fig. 3:**
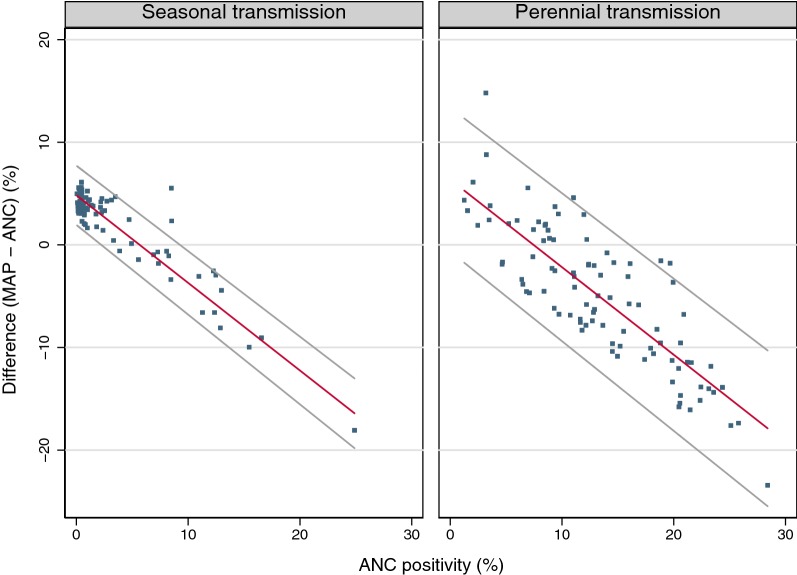
District test-positivity difference MAP − ANC against ANC malaria test-positivity with regression line and 95% limits of agreement

Comparing ANC test-positivity with 2015 estimates of the MAP, seasonality was the only significant covariate. Regardless of seasonality, the test-positivity difference decreased and eventually became negative with increasing ANC test-positivity (Fig. 3, Table 2). The curve for districts with perennial transmission was shifted to the right as compared to the curve for districts with seasonal transmission. The 95% limits of agreement were relatively narrow for districts with seasonal transmission and more than twice as wide for districts with perennial transmission. The width of the 95% limits of agreement interval did not change with increasing transmission levels.

#### Bayesian geo-statistical model fitted on MIS 2015/16

**Fig. 4:**
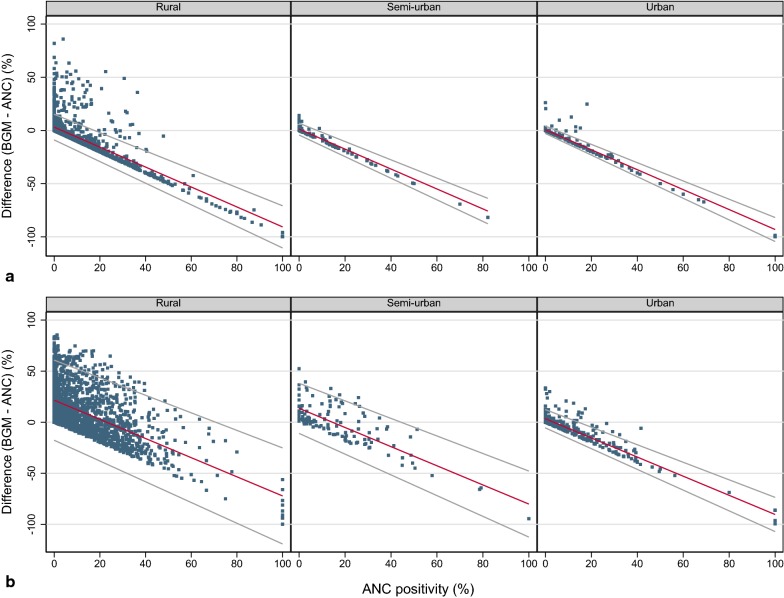
District test-positivity difference BGM − ANC against ANC malaria test-positivity with regression line and 95% limits of agreement. **a** Districts with seasonal malaria transmission, by district type. **b** Districts with perennial malaria transmission, by district type

The comparison of health facility-level ANC malaria test-positivity to modelled prevalence around health facilities showed similar results to the MAP comparison. While the test-positivity difference was positive at low ANC test-positivity, it eventually became negative with increasing ANC test-positivity. The level of urbanization affected the relationship between the ANC test-positivity and the modelled prevalence around health facilities in areas with perennial malaria transmission (Fig. 4b, Table 2). No effect was found for health facilities in areas of seasonal transmission (Fig. 4a, Table 2).

## Discussion

This is the first study to validate the use of large-scale routinely collected ANC malaria test-positivity data reported through a national health information system as an innovative and cost-effective approach for malaria surveillance at sub-national level. The analyses performed show that the malaria test-positivity in pregnant women attending ANC is related to other population and model-based malaria endemicity measures. Varying test-positivity differences between pregnant women and the comparison groups could be partially explained by different levels of seasonality, urbanization, and ITN usage. These findings suggest that ANC malaria test-positivity can be useful to assess community-level prevalence and sub-national heterogeneity of malaria endemicity. However, the variability of the test-positivity difference across the ANC test-positivity spectrum was high at all levels of comparison and could not be accounted for by the covariates that were used in these analyses.

Factors that will help to decrease the uncertainty are most likely to vary within the comparison groups and geographically. Travelling patterns, treatment-seeking behaviour or intervention coverage have been shown to vary in space and between different demographic groups with changing impact on their malaria prevalence [15–17]. However, the effect of ITN usage in this study was non-existent or too small to conclude an important influence of ITN coverage on the relationship between the malaria prevalence in children and the test-positivity in pregnant women and it might be rather seen as the consequence of increasing intervention coverage due to a high transmission level. Another factor previously suggested by van Eijk et al., and Hellewell et al. to increase the uncertainty of the relationship is gravidity [7, 8]. Gravidity is a direct consequence of fertility which differs with the level of urbanization and socioeconomic status which is likely to vary at sub-national levels [10]. Different levels of fertility determine the proportion of primi- and multigravidae women attending ANC. Van Eijk et al. showed that the malaria prevalence in primigravidae women compared to the prevalence in children provides results with less heterogeneity; thus, gravidity might have to be taken into account when using pregnant women attending ANC as a sentinel population for malaria surveillance [7]. However, routine ANC data in Tanzania so far does not provide information on gravidity on an aggregated level.

While factors that vary geographically and within the comparison groups can account for the variability found in this study, factors that differ between pregnant women and children are more likely to explain the mean difference at varying transmission intensities. First, the test-positivity in pregnant women might be lower than in children due to their higher capability to clear parasites from their blood after drug administration [18]. Secondly, infections in pregnant women may be less likely to be detected due to the sequestration of the parasite in the placenta and a higher level of immunity as the product of more cumulative exposure over years and during former pregnancies [7, 19]. However, earlier studies suggest that the protection against the *Plasmodium falciparum* malaria parasite does not merely depend on cumulatively acquired immunity but is also associated with the age of the host independent of previous exposure [20–24]. The reason might be constitutional differences between an adult’s immune system and the immune system of a child which potentially leads to a higher ability of the adult immune system to mount a protective response against the parasite infection [24]. The interaction between exposure and maturation of the immune system would particularly explain the comparatively high malaria prevalence in school children and the progressively decreasing relative difference between school children and pregnant women with increasing ANC test-positivity in areas of perennial malaria transmission. In settings with seasonal malaria however, the prevalence in school children was more comparable to the test-positivity in pregnant women than in areas with perennial transmission. This finding is likely to be caused by a comparatively low level of immunity in the adult population as malaria immunity has been shown to be short-lived and depending on constant exposure to the parasite, thus, fading during seasons of interrupted transmission [25, 26].

In contrast, seasonality of malaria transmission did not have a significant effect on the relationship between the malaria prevalence in children aged 6–59 months and the test-positivity in pregnant women attending ANC. However, the overall higher prevalence found by the TDHS-MIS 2015/16 still indicates that pregnant women most likely have a higher level of immunity than young children. The observed malaria prevalence in school children was considerably higher than in children 6–59 months. This finding confirms previous research that found older children to be less likely symptomatic than younger children while serving as a reservoir of malaria infection [27, 28]. A study by Walldorf et al. showed that in southern Malawi, school children were less likely to use bednets, were less often brought for treatment and often used unreliable treatment sources [27]. Additional to differences in immunity, these factors may explain a higher prevalence in older children compared to younger children.

Finally, this study found very different patterns for modelled prevalence data compared to survey data. The prevalence predicted by the MAP and the BGM was higher than the test-positivity in pregnant women up to a certain threshold but was consistently lower thereafter. It is difficult to find a biologically meaningful explanation for this finding.

The use of routine ANC test-positivity data in Tanzania is supported by high reporting rates and continuously increasing malaria testing rates. However, testing rates were lower in rural and semi-urban settings which tend to be less accessible than urban areas and might therefore be more prone to commodity stock-outs. Independent of district type, the ANC malaria testing rate was lower in districts with a malaria test-positivity above 5%. It can be hypothesized that in health facilities operating in areas of higher transmission symptomatic patients might be prioritized for malaria testing resulting in a shortage of RDTs at the ANC department. However, further investigations should be undertaken to gain better insights into the reasons for the difference in testing rates between areas of different levels of urbanization and malaria transmission.

This study was not without limitations. The direct comparison of routinely obtained ANC malaria test-positivity and data obtained from surveys and model predictions is complicated due to different methodologies. Selection bias is a potential factor influencing ANC malaria test-positivity in areas where testing rates are low. Additionally, routine data has previously been shown to suffer from data quality issues resulting in a need for thorough data cleaning which may by itself introduce biases [29]. Lastly, it is not clear how these findings for Tanzania can be transferred to other malaria-endemic countries. So far, ANC test-positivity is not a routine surveillance tool to monitor malaria transmission and no global guidelines exist on the most appropriate application of ANC test-positivity data. With ANC coverage being well above 90% in many sub-Saharan African countries (98% in Tanzania in 2015), and WHO recommending the use of routine information systems for malaria surveillance, this study provides support to the application of this approach on a wider scale despite a number of remaining uncertainties [6, 10, 30, 31].

## Conclusions

Since its introduction in 2013, routine screening of pregnant women attending ANC has made significant improvements in terms of coverage of malaria testing. This progress does not only provide increasing benefit to the target population by identifying and treating infected individuals but also contributes to reducing the reservoir of malaria transmission. This study shows that the relationship between routine ANC malaria test-positivity data and survey prevalence data in children follows expected patterns. However, the uncertainty of the relationship was high at all levels of transmission intensity as well as administrative levels indicating that ANC malaria test-positivity cannot be used to directly predict the prevalence in other population subgroups unless further research is undertaken to identify additional covariates that influence the relationship. Together with evidence from previous research, the findings of this study support the use of routine ANC malaria test-positivity data in combination with measurements in other age groups, to assess sub-national heterogeneity and trends of malaria in settings with representative ANC coverage. Consistent data collection over time may in the future offer a potential to use ANC malaria test-positivity to identify local foci of malaria transmission. Where foci appear stable over time, ANC malaria test-positivity can provide the basis for proactive case detection and targeted interventions, thus, contributing to the aim of malaria elimination [32].

## Supplementary information

**Additional file 1: Table S1.** Administrative regions of Tanzania stratified by geographic zone and malaria transmission**. Figure S1.** Flow chart for the number of reports of the ANC dataset included in the statistical analysis**. Figure S2.** Average malaria testing rate at ANC health facilities in districts with more and less than 5% ANC test-positivity, over time, by district type.

## Data Availability

Survey datasets used in this publication are publicly available from the referenced sources; ANC testing data are available from the Tanzanian National Malaria Control Programme (NMCP) but restrictions apply to the availability of these data, which were used under license for the current study, and so are not publicly available. Data are however available from the authors upon reasonable request and with permission of the Ministry of Health, Community Development, Gender, Elderly and Children of Tanzania.

## Abbreviations

ANC: antenatal care
BGM: Bayesian geo-statistical regression model
DHS: Demographic and Health Survey
DHIS2: District Health Information System 2
HMIS: Health Management Information System
ITN: insecticide-treated net
MAP: Malaria Atlas Project
MIS: Malaria Indicator Survey
RDT: malaria rapid diagnostic test
SMPS: school malaria parasitaemia survey
TDHS-MIS: Tanzania Demographic and Health Survey and Malaria Indicator Survey
WHO: World Health Organization
