# Supplementary material for: The potential of pregnant women as a sentinel population for malaria surveillance
Source: Malar J. 2019 Nov 21;18:370. doi: 10.1186/s12936-019-2999-0 (PMC6873723; doi:10.1186/s12936-019-2999-0)
Supplement: Supplementary file 1 — Additional file 1: Table S1. Administrative regions of Tanzania stratified by geographic zone and malaria transmission. Figure S1. Flow chart for the number of reports of the ANC dataset included in the statistical analysis. Figure S2. Average malaria testing rate at ANC health facilities in districts with more and less than 5% ANC test-positivity, over time, by district type. [file 12936_2019_2999_MOESM1_ESM.docx]

Additional file 1

**Table S1: Administrative regions of Tanzania stratified by geographic zone and malaria transmission.**

| Region | Geographic zone* | Malaria transmission** |
| --- | --- | --- |
| Dodoma | Central | Seasonal |
| Manyara |  |  |
| Singida |  |  |
| Arusha | Northern |  |
| Kilimanjaro |  |  |
| Tanga |  |  |
| Iringa | Southern Highlands |  |
| Njombe |  |  |
| Ruvuma |  |  |
| Katavi | Southwest Highlands |  |
| Mbeya |  |  |
| Rukwa |  |  |
| Dar Es Salaam | Eastern | Perennial |
| Morogoro |  |  |
| Pwani |  |  |
| Geita | Lake |  |
| Kagera |  |  |
| Mara |  |  |
| Mwanza |  |  |
| Shinyanga |  |  |
| Simiyu |  |  |
| Kigoma | Western |  |
| Tabora |  |  |
| Lindi | Southern |  |
| Mtwara |  |  |

*****Tanzania Demographic and Health Survey and Malaria Indicator Survey 2015/16

** Progress and Impact Series – Focus on Mainland Tanzania, WHO 2012

**
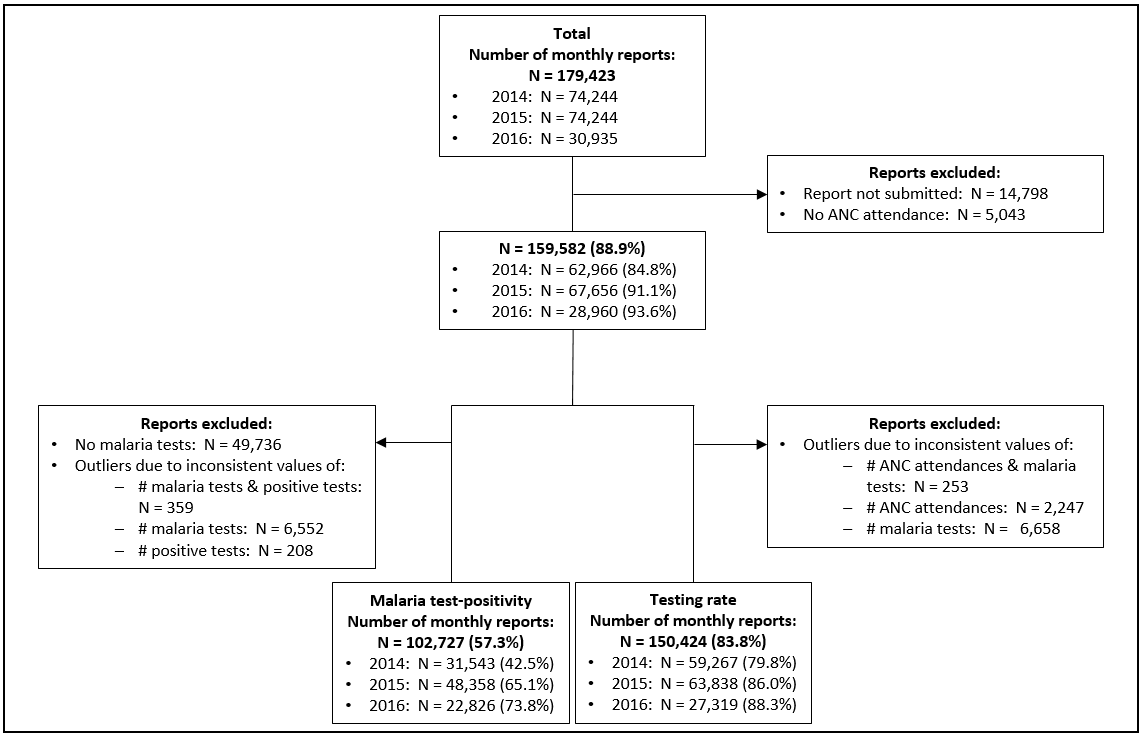
Figure S1: Flow chart for the number of observations of the ANC dataset included in the analysis.**


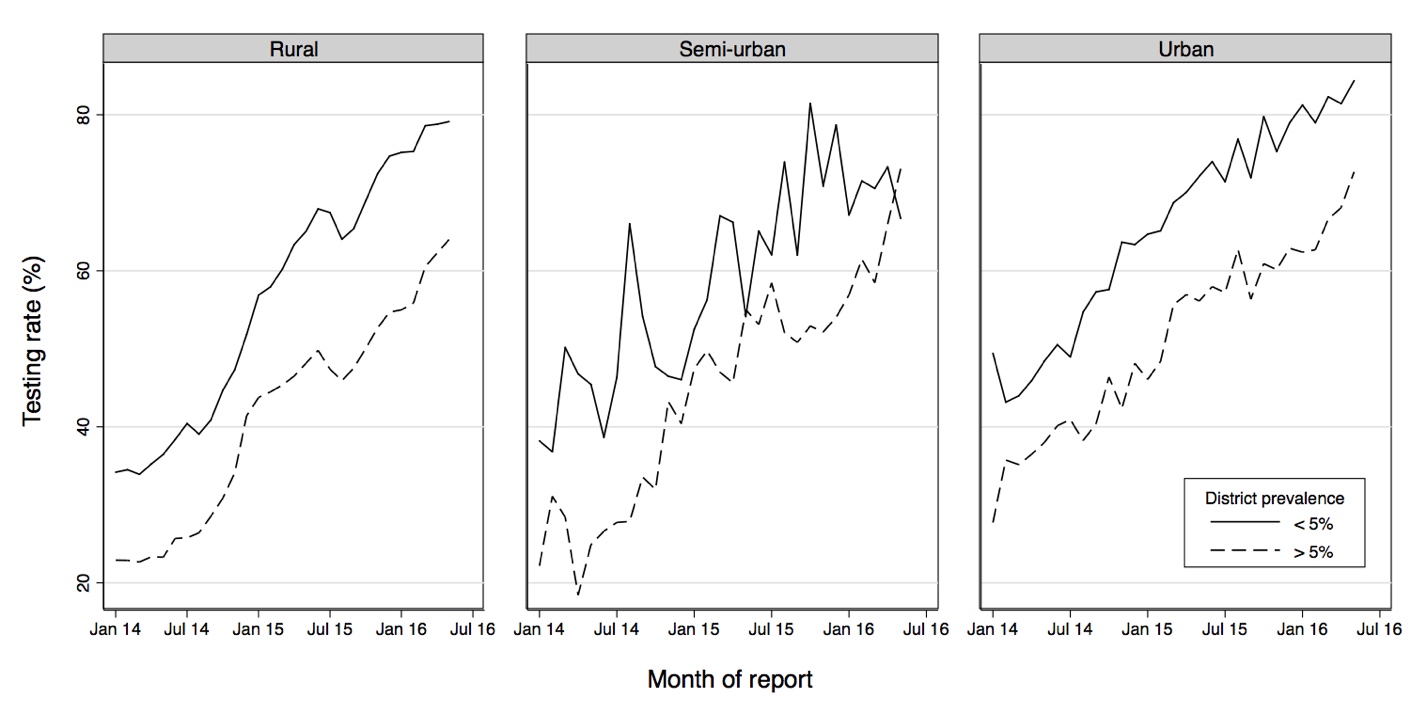


**Figure S2: Average malaria testing rate at ANC health facilities in districts with more and less than 5% ANC test-positivity, over time, by district type (rural, semi-urban, urban).**
